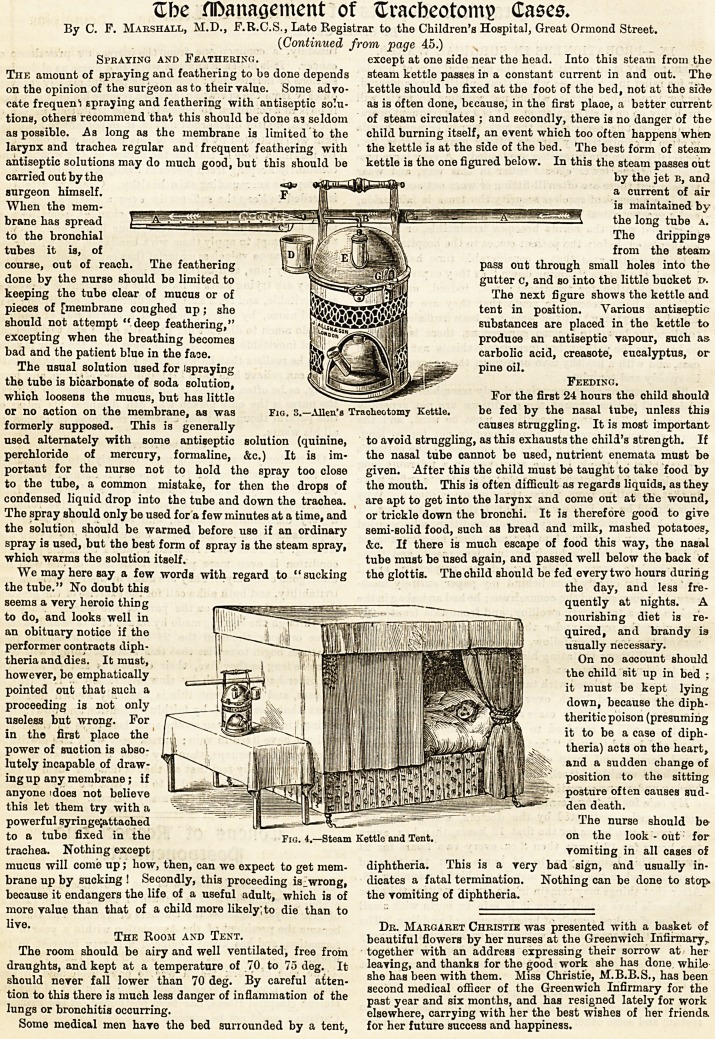# "The Hospital" Nursing Mirror

**Published:** 1897-11-06

**Authors:** 


### "The Hospital" Nursing Mirror.

Being the Nursing Section of "The Hospital."

[Contributions for this Section of "The Hospital" should be addressed to the Editor, The Hospital, 28 & 29, Southampton Street, Strand, London, W.C., and should have the word "Nursing" plainly written in left-hand top corner of the envelope.]

### News from the Nursing World.

### IN MEMORIAM.

Amidst all the trapping of woe the mortal remains of the Duchess of Teck were laid to rest at St. George's Chapel, Windsor, on Wednesday, November 3rd, at one p.m. Memorial services were held in the Chapel Royal, St. James's, and at St. Paul's Cathedral. The City authorities caused all traffic to be stopped round the Cathedral, so that nothing broke the silence but the tolling of the knell. Hushing the busy City to solemn stillness in the middle of the day expressed the universal sorrow felt for the beloved and honoured dead in a most appropriate and dignified manner.

### TYPHOID AT UNIVERSITY COLLEGE HOSPITAL.

We are glad to be able to announce that the outbreak of typhoid at University College Hospital is stamped out, and that, in spite of reports to the contrary, no death has taken place amongst those attacked. All excepting two, who are in a critical but not hopeless condition, are out of danger, and some have reached the "custard stage" of recovery. It is true that a pensioned nurse of the staff died last week, but it was not of fever, and not at the hospital. This nurse had been on the staff for 26 years, and was on a visit to her brother at St. Mary Cray. She suddenly fell ill, and died on the day before her intended return to London.

### THE NURSES' HOSTEL "AT HOME."

The manager and residents of the Nurses' Hostel are comfortably settled in their new quarters in Francis Street, and a house warming in shape of an "At Home" on November 1st was largely attended. The really beautiful hall and staircase showed to great advantage, and the whole building was brilliant with electric light. The nurses are very proud of their new premises, and played the part of hostesses cordially. Miss Wood has evidently the arts of understanding her guests and of inspiring them with a feeling of freedom. Judging by their animated countenances and conversation both hostesses and guests amused themselves thoroughly.

### BAZAAR AT HACKNEY.

A successful bazaar in aid of the North-Eastern Hospital for Children, Hackney Road, was completed yesterday at the Hackney Parish Room. Lady Elizabeth Biddulph being unable to attend in consequence of the death of the Duchess of Teck, the opening ceremony of the second day (Thursday) was performed by Mrs. Herbert Robertson, wife of the member for South Hackney. In an eloquent appeal for the Building Fund the Rev. W. G. Morcom stated that 700 children were received into the wards, and that 16,000 were treated in the out-patient department annually, the latter making 55,000 attendances, but that each year at least 600 cases requiring in-patient treatment were refused admission to the wards for want of room, and that the hospital, although now doing a splendid work, was hampered in every way by the want of proper accommodation. The present building contained only 57 beds, and the completing wing, left unfinished since 1880, would provide probably another 50.

### SUCCESS.

The Guardians of Chelsea Infirmary have successfully carried their point, and have received permission from the Local Government Board to give Miss Norrie, the young nurse who contracted consumption in the discharge of her duties, the sum of £50. It will be remembered that this is the sum originally voted, and to which the Local Government Board demurred. The Guardians, however, were determined that the nurse should have it even if they paid it themselves. They have therefore represented the matter so strongly at headquarters that the authorities have reconsidered their decision and given their approval.

### YARMOUTH BLUNDERERS.

The mismanagement at Yarmouth Workhouse reached a climax when the other week 200 sick were consigned to the sole charge of two assistant nurses and a probationer of six months' standing. A committee of three lady and three men guardians has been appointed by the board to take the matter in hand, and it is to be hoped that they will institute thoroughgoing reforms with a strong hand.

### A BISHOP'S NURSE.

Dr. Sheehan, Bishop of Waterford, has taken a step with regard to nursing in Irish infirmaries that must win universal approval. He proposes to send to Clonmel Hospital a trained nurse from London, who will instruct the nuns and attendants in nursing, and he offers to bear the expense himself. The Guardians have accepted the Bishop's offer. This is a very practical endeavour to solve a vexed question, for the objection to the employment of nuns for nursing paupers is removed when they are properly instructed in their work.

### VICTORIA NURSES' HOME, OTLEY.

On Saturday, October 23rd, a nurses' home was opened by Mr. Barker at Westgate, Otley, Leeds, in commemoration of this memorable year now fast drawing to a close. A nurse, Miss Annie Nesbit, began work in the district some nine months ago, and the home will now afford accommodation for several patients as well as the nursing staff. Mr. Bell, the secretary, received in recognition of his services a golden chain, which Mrs. Fawkes presented on behalf of the committee.

### STOCKPORT NURSING ASSOCIATION.

The Stockport Sick Poor Nursing Association is a useful institution, and by the amount of really hard work accomplished and the excellent way in which it appears to be managed fully merits the affection and support given to it. Whilst the steady work of the nurses continues unchecked, the committee are seeking to enlarge its borders in four ways as a memorial of the present noteworthy year. They have as yet only begun with one, and that is the sending of convalescent patients away for change. No steps will be taken just at present to erect a nurses' home, as there are other charities in the town claiming attention, but this will undoubtedly come in time, as the nurses' services are highly appreciated amongst the working classes, a fact shown by an amount of £6 being subscribed by grateful patients.

### CARLISLE DISTRICT NURSING ASSOCIATION.

SEVERAL changes are recorded in the sixth annual report of this association. The lady superintendent for the Denton Holme District has resigned, and her place has been taken by Miss Musgrove. Nurse Bates has accepted work elsewhere, and Nurse Keay has not been well enough to work. It is satisfactory to note from the report how firmly the nurses are established in the affections of the district. The number of cases has increased so much that additional help has had to be engaged. Patients who can afford it pay for the nurses' visits.

### ESSEX NURSES FOR ESSEX.

OF late years it has been the fashion to endeavour to narrow the limits within which a person may work. In accordance with this principle the Committee of the Essex County Cottage Nursing Association patriotically declare that Essex women alone should be trained for that county. The fate of "many a well-laid scheme" has fallen on this plan, and it "has 'gon aglee" because the women of Essex will not come forward in sufficient numbers to supply the demand. Now the County Cottage Nursing Association throws its gates wide open, and invites applications from outside, provided the candidates promise a specified term of service in Essex.

### A DUAL NOVITIATE.

NURSING from time immemorial has been the especial task of religious communities. Within the last few years the standard of the work has risen so greatly, and lay nurses, trained under the instruction of scientists, have so surpassed the achievements of religious sisterhoods, that the latter now lie under all the disadvantages of antiquated methods and a dual novitiate. The difficulty of this dual training has been well illustrated in the case of an Anglican religious community who for some time have been engaged in the care of incurables. The Home is in London. A few months ago the Mother Superior, a skilled, capable, trained nurse, moved into a more commodious residence, and placed the nursing into the hands of two trained lay charge nurses, and confined the sphere of the mistress of the novices—who had hitherto ruled all—to the religious side of their work. A dual authority was thereby established which occasioned considerable friction. The charge nurse, loyally supported by the Mother Superior, had to face much annoyance from her probationers' neglect of their secular duties in order to attend to their religious exercises. On one occasion, a probationer in the midst of performing the toilet of a patient, left her in order to fetch something that had been forgotten. Whilst she was out of the room the bell for "Tierce" rang, and the pious probationer forsook the incurable and all else, and hastened to her devotions without considering it needful to inform her secular superiors in the ward of her intention. The poor patient was discovered in a comfortless state of waiting. There are one or two points here which give rise to necessary discussion. The religion of a nurse is a thing with which we have nothing to do, but the training of a nurse is a very different matter. The step of the Mother Superior in appointing a trained charge nurse over the ward is in the right direction, for unless the nursing of the sisterhoods meets the requirements of the day, this employment must of necessity pass into other hands. To attain proficiency a probationer must give her whole time and attention to her work; this is impossible if she has to spend a great part of her day in church. The dual novitiate at the same time is then impracticable, and it remains for the heads of religious communities to consider their position and rearrange the duties of their novices, so that a patient need not remain half-dressed whilst her nurse says her prayers.

### SHORT ITEMS.

A REPORT of the eleventh year's work and financial affairs of the Barton-on-Humber District Nursing Association shows that the sick are cared for as well as nursed. Patients are encouraged to pay what they can afford, and instruction is given to members of their own family in the art of sick cookery.—Mr. William Lees, of Townlane, Denton, has promised £500 to build a Nurses' Home for the nurses of the new association, to be founded in commemoration of the present year, provided subscriptions for the support of two nurses come in. These already amount to £140.—The Jubilee County Memorial Fund for Bedford amounts to £1,200. It is to remain open until the end of the year, as another £500 or £600 is needed to cover the expenses of erecting a Nurses' Home in connection with the County Hospital. The name of the new Home is "The Victoria Nurses Home of the Bedford County Hospital," and the plans and contracts have already been accepted. —The Sisters of Mercy, in whose care lies the nursing of Limerick Workhouse Hospital, have received a warm testimony, both as regards the method and efficiency of their work, and as to their devotion and attention to the sick and aged. The letter reporting the condition of the nursing to the governors is signed by the resident and visiting medical surgeons.—After a spirited discussion, St. George's Board of Guardians voted Miss Wesley, the matron, a gratuity of £20 in recognition of her services. She has improved the nursing in the infirmary greatly, and in training nurses has saved considerable outlay.—The assistant matron of Mile End Infirmary informs us that the person charged with cruelty towards the children in her care, and asked to resign, was not a sick nurse, nor attached to the infirmary staff. She was an untrained attendant only. —Miss Jackson, the lady selected by Miss Peter, Q.V.J.I., as district nurse of Torpoint, takes up her new work on November 15th.—The trust deed of Victoria Nursing Institute, Gosport, has been signed. The Princess Henry of Battenberg is to be asked to become president.—On Friday evening, October 29th, an excellent concert was given by "The Dighton Trio" and friends to the patients and nurses at the Consumption Hospital, Mount Vernon, Hampstead, N.W. The programme was well received, and the artistes cordially applauded.

### Practical Aspects of a Nurse's Life.

By Sister Grace.

### IX.—PROBATIONERS IN SURGICAL WARDS.

Herniotomy is an operation for the practical cure of hernia, or for relief in acute cases where strangulation has appeared, or for irreducible hernia, which, not yielding to taxis, will probably go on to strangulation. It is a very frequent operation in hospitals, especially in the male wards, because hernia is often precipitated by strains, lifting heavy weights, &c., especially when the intestines are loaded. A very large proportion of the lower classes suffer in this way, and wear trusses, but these are often ill-fitting or worn out and useless, and in a moment of careless security the truss is laid aside, and a greater stress than usual being put upon the feeble abdominal wall, the hernia becomes irreducible, or perhaps strangulated, before the patient comes to the hospital.

It is often the case that a considerable time has elapsed between the hernia "coming down," as they express it, and their application for treatment; not, indeed, until their sufferings have become so acute that they are willing to submit to anything. If the time has been sufficiently long to allow the bowel to become gangrenous, there is little or no hope for the patient, but fortunately this is not often the case, and with a healthy man or woman the recovery should be speedy and complete.

The treatment after operation closely resembles that after abdominal section, though the conditions are not generally so serious. The same precautions must be used, and the same vigilance exercised, though happily not for so long. The same difficulty will arise in consequence of the patient's great thirst; I think I have remarked in a tolerably long experience of herniotomy cases that the patients suffer more in that operation from thirst than in many others. Why this should be so I know not, but I think it is a fact. I remember one man in a very critical condition, who wearied me and every one round him for more than his allowance of drink, until suddenly his importunities ceased, and there was absolute peace. This aroused my suspicions, and I watched him closely from behind my paper entirely unobserved. I soon solved the conundrum; he had an icebag in the bed to ease a temporary swelling, and from this (cautiously removing the screw under the bedclothes) he drank, and sucked the ice. Poor fellow, that comfort was soon withdrawn. Without irritating him by any remark, I removed the legitimate icebag, and substituted one made of gutta-percha tissue and closed with turpentine. When he recovered he enjoyed a good laugh over sister being what he called "a deep 'un." Hernia cases are nursed in the ward, and great care will be required to see that friends do not bring in what I heard termed "just a snack," which consisted of a red herring and lump of Dutch cheese, which the wife considered would be "relishing" for her husband's tea, he having undergone operation the day before.

My rule for dieting hernia cases after operation, if I was not otherwise instructed by the doctors, was as follows: ½ oz. every two hours for the first 12 hours, then 1 oz. every two hours for 12 hours, then 2 oz. every two hours for 24 hours. This brings you to 48 hours from the time of operation, when the amount may be doubled for another 48. After that things may remain in the same condition till after the bowels have acted, when it is safe to begin with small portions of soaked crumb of bread or custard pudding. The bowels will not act probably without an enema; at first be very gentle and slow in its administration. I hope it is unnecessary at this stage of your nursing education to warn you not to pump in air.

### Colotomy.

This operation, for the relief of malignant disease of the rectum and some other forms of obstruction, is also, unfortunately, a common one from the increasing prevalence of cancer. It is not such anxious work to a nurse as abdominal sections or herniotomy; indeed, the patient usually recovers quickly from the operation, and can resume his ordinary food if he is in a condition to enjoy it. The irritating nature of the fæcal matter discharged makes it important to keep the skin well protected by a piece of lint covered with ointment. By making a hole in this for the orifice of the wound, and constantly keeping fresh pieces at hand, it is quite possible to keep the surrounding skin healthy. Constant attention is needed to keep the sufferer in even a fairly sweet condition; the dressings require constant changes, sometimes as often as eighteen times in twenty-four hours, and it is well not to attempt to apply them with bandages, but with broad binders of some valueless material that can be fastened easily with safety-pins, and burnt after use.

They are trying cases to nurse, for the patient is depressed and irritable, and feels keenly his distressing condition; but a good nurse, by cheerfulness and a bright way of talking, may do much to distract her patient's mind and to dispel the almost inevitable gloom that descends upon the poor invalid when he realises that he will never be better, and that death alone can relieve him from his humiliating position, and from being, as he often feels he is, an object of disgust to others; the nurse is the only person who can disabuse his mind of this painful thought by her cheerful readiness to attend to all his wants.

### Gastrostomy.

Gastrostomy is an opening made directly into the stomach for the administration of food when the natural passage is closed either by malignant disease or some other cause. Like colotomy, it cannot be more than an effort to give relief to a very distressing condition, and one's knowledge of the hopelessness of ultimate benefit makes such cases very distressing. It is not often resorted to, and I think the patient's condition is even more pitiable than that of one who has undergone colotomy. They are alike in their depression and irritability, and both alike call for the exercise of the greatest devotion and patience on the part of the nurse. They are fed through the opening made by means of a tube and funnel, three or four times in the 24 hours, and when the poor creatures begin to realise that this state of things will continue as long as they live, their despair is sad to see. On the other hand, a death by slow starvation is frightful to witness, and at present this is the only known means to avert that death. If by faithful attention and unwearied, loving kindness you can soothe such last days as these you will not have lived in vain; nurses meet with less gratitude in proportion, perhaps, than any other body of women, for sufferers generally feel that all that is done for them is only their right; but our reward is to feel that we have soothed much suffering and comforted many sorrows.

### Tokens of Respect and Postponements.

The Mary Adelaide nurses of the Workhouse Infirmary Nursing Association sent a wreath of flowers to the funeral of Her Royal Highness the Duchess of Teck. The Duchess became the president of the Association within a year of its formation, and has always evinced a hearty interest in its work.

The sale of work which was to have been held in the Jerusalem Chamber, Westminster Abbey, in aid of the St. Helena Hospital Home, has been postponed, as the Princess Christian could not be present.

### The Management of Tracheotomy Cases.

By C. F. MARSHALL, M.D., F.R.C.S., Late Registrar to the Children's Hospital, Great Ormond Street.

(*Continued from page* 45.)

SPRAYING AND FEATHERING.

THE amount of spraying and feathering to be done depends on the opinion of the surgeon as to their value. Some advocate frequent spraying and feathering with antiseptic solutions, others recommend that this should be done as seldom as possible. As long as the membrane is limited to the larynx and trachea regular and frequent feathering with antiseptic solutions may do much good, but this should be carried out by the surgeon himself. When the membrane has spread to the bronchial tubes it is, of course, out of reach. The feathering done by the nurse should be limited to keeping the tube clear of mucus or of pieces of membrane coughed up; she should not attempt "deep feathering," excepting when the breathing becomes bad and the patient blue in the face.

The usual solution used for spraying the tube is bicarbonate of soda solution, which loosens the mucus, but has little or no action on the membrane, as was formerly supposed. This is generally used alternately with some antiseptic solution (quinine, perchloride of mercury, formaline, &c.) It is important for the nurse not to hold the spray too close to the tube, a common mistake, for then the drops of condensed liquid drop into the tube and down the trachea. The spray should only be used for a few minutes at a time, and the solution should be warmed before use if an ordinary spray is used, but the best form of spray is the steam spray, which warms the solution itself.

FIG. 3.—Allen's Tracheotomy Kettle.

We may here say a few words with regard to "sucking the tube." No doubt this seems a very heroic thing to do, and looks well in an obituary notice if the performer contracts diphtheria and dies. It must, however, be emphatically pointed out that such a proceeding is not only useless but wrong. For in the first place the power of suction is absolutely incapable of drawing up any membrane; if anyone does not believe this let them try with a powerful syringe attached to a tube fixed in the trachea. Nothing except mucus will come up; how, then, can we expect to get membrane up by sucking! Secondly, this proceeding is wrong, because it endangers the life of a useful adult, which is of more value than that of a child more likely to die than to live.

THE ROOM AND TENT.

The room should be airy and well ventilated, free from draughts, and kept at a temperature of 70 to 75 deg. It should never fall lower than 70 deg. By careful attention to this there is much less danger of inflammation of the lungs or bronchitis occurring.

Some medical men have the bed surrounded by a tent, except at one side near the head. Into this steam from the steam kettle passes in a constant current in and out. The kettle should be fixed at the foot of the bed, not at the side as is often done, because, in the first place, a better current of steam circulates; and secondly, there is no danger of the child burning itself, an event which too often happens when the kettle is at the side of the bed. The best form of steam kettle is the one figured below. In this the steam passes out by the jet B, and a current of air is maintained by the long tube A. The drippings from the steam pass out through small holes into the gutter C, and so into the little bucket D.

The next figure shows the kettle and tent in position. Various antiseptic substances are placed in the kettle to produce an antiseptic vapour, such as carbolic acid, creasote, eucalyptus, or pine oil.

FEEDING.

For the first 24 hours the child should be fed by the nasal tube, unless this causes struggling. It is most important to avoid struggling, as this exhausts the child's strength. If the nasal tube cannot be used, nutrient enemata must be given. After this the child must be taught to take food by the mouth. This is often difficult as regards liquids, as they are apt to get into the larynx and come out at the wound, or trickle down the bronchi. It is therefore good to give semi-solid food, such as bread and milk, mashed potatoes, &c. If there is much escape of food this way, the nasal tube must be used again, and passed well below the back of the glottis. The child should be fed every two hours during the day, and less frequently at nights. A nourishing diet is required, and brandy is usually necessary.

FIG. 4.—Steam Kettle and Tent.

On no account should the child sit up in bed; it must be kept lying down, because the diphtheritic poison (presuming it to be a case of diphtheria) acts on the heart, and a sudden change of position to the sitting posture often causes sudden death.

The nurse should be on the look-out for vomiting in all cases of diphtheria. This is a very bad sign, and usually indicates a fatal termination. Nothing can be done to stop the vomiting of diphtheria.

---

DR. MARGARET CHRISTIE was presented with a basket of beautiful flowers by her nurses at the Greenwich Infirmary, together with an address expressing their sorrow at her leaving, and thanks for the good work she has done while she has been with them. Miss Christie, M.B.B.S., has been second medical officer of the Greenwich Infirmary for the past year and six months, and has resigned lately for work elsewhere, carrying with her the best wishes of her friends for her future success and happiness.

### Funeral Customs in America.

THE CLOTHING OF THE DEAD.

Perhaps in no particular is the dissimilarity of English and American funeral customs marked so strongly as in the matter of dressing the dead.

A marked change has come over English funerals, so far as profuseness of flowers and wreaths is concerned, but we still commit our dead to Mother Earth shrouded in simple white garments—more or less lace-trimmed according to sex and worldly circumstances. A few white flowers placed in the hands or laid on the breast are the only concessions to decorative attempt, while the hair falls into the kinks and waves natural to life, or is smoothly combed and plainly parted. So that the duties of the nurse are very simple.

In the United States it is customary to bestow much skill and attention on the preparation of the dead, and frequently not only the aid of the embalmer, but the services of the hairdresser and costumier are requisitioned. Undertakers in New York pay as much attention to the cut and fashion of "burial robes," as does a Regent Street costumier to the worldly trappings of critical clients still in the flesh. To this end each undertaker of any standing has in his regular employ one or two dressmakers for the dead, who are trained to this work and devote all their energies to designing burial robes.

It is quite customary, too, for undertaking firms to include in their advertisements and catalogues of funeral furnishings an engraved supplement, showing an infinite variety of death costumes both for men and women. This is by no means unlike a fashion plate, and contains examples of "burial clothing" which may cause a little shock at first sight to English eyes a customed as these are to the simple white gowns in which we clothe our dead. It must be confessed that our simplicity is more reverential, for however pretty an accordion pleated chiffon bodice with full puffed sleeves may be on the living it is not an ideal costume in the coffin. Unfortunately, too, corsets for women corpses are by no means unusual, though, needless to say, in such a case no undue construction would be employed. Many a young girl in America goes down to the grave in the dress designed for her début at a ball, complete to fan, feathers, and elaborate coiffeur. For the services of a hair-dresser are frequently called into requisition to complete the offices of a death dressmaker. And some manicures in New York derive a fair proportion of their income from polishing and manicuring the nails of the dead—the rate of pay for this being considerably higher than a similar service performed for a live client.

An English nurse must feel that many of her traditions are injured when she helps to clothe a dead person in a modern costume, while she must at the same time confess that a young girl in her coffin wearing her Communion robes is a distinctly pretty sight. And though it is proverbial that many a man shows to the best advantage in evening dress, this attire looks distinctly unsuitable to the background of a coffin. To the American mind, however, there is nothing unsuitable—in fact, to them it seems a mark of honour to the deceased to clothe him in a new dress suit, with all the added niceties of jewelled studs, faultless white tie, and patent leather pumps.

To be a successful funeral dressmaker much practice and skill is necessary, for many of these grave garments have no "backs" to them. Consisting in the majority of cases of "fronts" only, they are made so as to give to the body lying in the coffin all the appearance of being fully dressed, and by a skilful system of lacing at the back, a good deal of the disturbance consequent on adjusting these elaborate toilettes for the dead is avoided.

### Temporary Nursing Arrangements at University College Hospital.

Thanks to the kindness of the Sister Superintendent, we have been able to inspect the temporary quarters to be occupied by the nursing staff of University College Hospital until the happy day when they will be removed wholesale to the nursing block of the new hospital now in process of building. Two children's wards and a small obstetric ward have been fitted up as dining hall, sitting-room, and cloak-room, whilst one angle with a window is converted into a very cosy corner indeed. Dinner was laid at the time of our visit, the tables were well arranged, the linen clean and uncrumpled, and the flowers fresh. The bill of fare consisted of joint, meat pasties, cabbage, potatoes, apple tart, bread and cheese. Ale and stout were served as wished, wine at the order of medical officer. Probationers and nurses are allowed to have wine sent by relatives, but it is given into the charge of the housekeeping sister, and is served only at meals. Such a precaution is eminently desirable. The sitting-room is most comfortable and pretty. Tables of different shapes and sizes, easy chairs, couches, screens, a piano, books, and plenty of air and light everywhere.

It is not probable that another outbreak of typhoid will occur. In the kitchen a Berkefeld filter has been fitted and every imaginary precaution taken. The servants' dining-room opposite is light, airy, and comfortable.

There is a unique arrangement of picnic baskets for the night nurses. The housekeeping sister packs daily either ham, eggs, or meat, tea, coffee, or cocoa into a small square basket. Each night nurse when she inspects her basket changes whatever she does not like for something she does. For instance, if she prefer eggs to the meat, or cocoa to the coffee that has been packed for her, she can have it changed. Dinner is ready for the night nurses at nine a.m. They can then take exercise or rest as they wish, but a minimum of seven hours' sleep is insisted on. Tea is carried round to them at half-past four. If a nurse wants to remain undisturbed she leaves a note to this effect outside her door and her wish is respected.

We are informed that there are no restrictions as to religious creed amongst the nurses and probationers on the hospital staff. If a nurse does not wish to attend prayers, she has only to ask permission of the sister superior, and it is always granted. But, for the sake of discipline, nurses not attending prayers must retire to their own rooms. One difficulty no amount of ingenuity can overcome, viz., the arrangements that have had to be made for providing sleeping accommodation in different houses. The passing to and fro between the hospital and the rooms is undesirable in many ways, but the only thing to be done has been done, viz., to make the best of it. Great pains have been spent on the sleeping rooms, and the ample cloak-room in the hospital gives every facility for changing damp clothing.

### Death in Our Ranks.

A death is, perhaps, never more sad than when associated with duty as its cause, and the pathos is enhanced when that duty is ministering to the sufferings of another. It is, therefore, with regret that we have to record the death of Nurse M. A. Dawson, of the St. George's-in-the East Infirmary, on October 28th, from enteric fever, contracted whilst nursing a case of that disease—the patient being an old woman who had pneumonia as a complication, and yet she made a good recovery; whereas the nurse, a young woman, has succumbed to her attack. Nurse Dawson was trained at St. George's-in-the-East, and was a kind, capable, and conscientious nurse. At a meeting of the Board of Guardians a vote of condolence with her relatives was passed.

### Workhouse Infirmary Nursing Association.

At a meeting of the Executive Committee held at the house of Lady Wantage on Wednesday, October 13th, the following resolutions were passed: "That the Executive Committee, after long and careful consideration, is of opinion that the time has arrived when the work of the Association must of necessity cease, for the following reasons: The impossibility of adequately meeting, by private effort alone, the demand for trained nurses in Poor-law infirmaries, under the prevailing conditions, viz.: (*a*) Inadequate financial support to meet the expenses of training; (*b*) lack of suitable candidates for training; (*c*) the anomalous position of trained nurses in country workhouses. The committee consider that steps should be taken to urge upon the Local Government Board that the whole question of nursing in Poor Law infirmaries should be undertaken by a State Department, or should be the subject of a Departmental inquiry." These resolutions were moved by the Hon. Mrs. J. Talbot and seconded by Mrs. Bonham-Carter. Miss Louisa Twining moved a resolution, "That a meeting should be called in order that the views of the Executive Committee should be laid before the General Committee and the subscribers," which was seconded by Mrs. A. C. Powell. A meeting is to be held on December 2nd, at three o'clock, in St. Martin's Town Hall, to consider the views expressed by the Executive Committee.

It is with much regret that we publish this decision. At no time were the offices of the Workhouse Nursing Association more wanted than at present. The Local Government Board have issued new regulations with a view of securing better nursing for the poor in workhouse infirmaries. These new regulations have suddenly created a demand which it will be difficult to supply. Guardians have never impressed us as being especially gifted in selecting candidates for their nursing staff, and now that it is determined that the standard of workhouse nursing shall be raised, then difficulties will be greater. When the Workhouse Nursing Association no longer exists they will have to rely on their own judgment, and if we may judge by one example, an exodus of the incompletely-trained from the hospitals to the union infirmaries is not improbable. We quote from the report of a meeting of the Bath Board of Guardians to show what training is necessary to secure a workhouse appointment." Nurse A. S., from the Holborn Workhouse, Mitcham, and Nurse A. S., from the Queen's Jubilee Hospital, were the candidates before the board. They had both had hospital experience, but neither had been long enough at their respective places to obtain certificates. Both were deemed efficient, and were appointed at a salary of £20." It would, therefore, appear that probationers in institutions, which cannot be regarded as training schools, can obtain after a short experience £20 per annum, whereas in a large hospital they receive either a very small sum or nothing—and in some cases even pay—during their period of training. What a premium on inefficiency is here revealed! For years past the Workhouse Nursing Association has not only been of the greatest assistance in assisting guardians in selecting candidates, but has supplied a large number of nurses properly trained for workhouse nursing. At the present time the whole standard of training in workhouse infirmaries runs the danger of being lowered. The best source of obtaining nurses is possibly to be removed, and workhouse nursing may be very little better than before the new regulations were issued. We feel that a strong appeal should be made to those interested in the welfare of an exceedingly helpless class of the community—the sick pauper—not only to make the continuance of the Workhouse Nursing Association possible, but to enable it greatly to extend its most useful work.

### Nursing and Other Notes from Maidstone.

Two more emergency hospitals have been opened at Maidstone. They are to be used only for the reception of patients awaiting removal to convalescent homes, and who, having passed the acute stage of typhoid fever, are still needing great care.

There is no perceptible diminution in the number of trained nurses in the town, and the night nursing of the sick in their own homes is receiving more attention.

Nurses from London and other institutions are deputed to this responsible work, and there is a night superintendent. Trained nurses (not connected with any special institution) are also doing voluntary work amongst the poor, which is duly organised in the parishes. There also appear to be untrained persons engaged in one or two quarters for night duty. It seems impossible for the general public to understand that the sick frequently require more care and equally skilled attendance during the lonely night watches than in the daytime, when doctors are more easily summoned and the patient's own friends are alert.

The laundry, which has been largely added to, and is now of immense service to private families as well as the hospital, stands in an airy, open spot near the permanent isolation hospital. The soiled linen is received daily, being collected in special carts, and brought in labelled bags from the town. Everything is steeped in disinfectants, boiled, washed, mangled, and aired before being returned to the owners.

It is satisfactory to learn that none of the doctors have contracted typhoid, and it is to be hoped that their immunity will continue. The only nurse who has had it so far is doing very well, and is very well cared for at the pleasant West Kent Hospital. Another nurse who has been more seriously ill (but not with typhoid) has improved very much within the last few days, and will probably soon be reckoned convalescent.

A house has been prepared, with thoughtful kindness, for the reception of invalid nurses, but it has so far not been occupied. It must be better for those who require a few days off duty to be together and well looked after in this private house rather than scattered in solitary rooms in various quarters. Under the latter circumstances no regular care and attention can be satisfactorily secured for the incapacitated workers. The kind feeling evinced in Maidstone towards the emergency nurses seems to increase rather than diminish, and the cordial good fellowship amongst the workers is also a pleasant feature in a trying experience.

"The Nurses' Stores" form a common meeting ground for all employed among the sick, and every day groups of busy nurses swarm in and out, bearing away great parcels of "comforts" for their patients. The support which has been given in every part of England to "the Stores" has been far beyond any expectations of their promoter.

Yorkshire women (workers themselves) have sent generous gifts of clothing. Many needlework societies have forwarded noble bales. Children in schools have given themselves, and collected from their friends, great sacks of toys, books, and pictures; and many letters have come expressing sympathy and kindness. Some of the offerings have a sad pathos about them, for bereaved mothers have given clothes and toys long treasured for the sake of their dead children. In the face of the present widespread sickness many a sacrifice of personal feeling is made, and many of the gifts received at the Nurses' Stores, Maidstone, bear evidence of generous feeling and of self-denial in the senders.

### Everybody's Opinion.

[Correspondence on all subjects is invited, but we cannot in any way be responsible for the opinions expressed by our correspondents. No communication can be entertained if the name and address of the correspondent is not given, or unless one side of the paper only is written on.]

### BREAKING CONTRACTS.

M. JOHNSON writes: As a nurse I would suggest in extenuation of the facts of "Breaking Contracts" that committees and honorary secretaries might be quicker in their selection of candidates for advertised posts. I know by personal experience that ten days and more will elapse before any notice is taken of the application. In these days of keen competition and greater percentage of candidates to vacancies, fifty or more nurses are kept in suspense, and some meanwhile accept unsuitable posts for fear of being "out of employ," which is a serious evil to many of us.

[*⁎* This correspondent is mistaken in supposing that an application for a post constitutes a contract. What Mr. Vincent Jackson rightly objects to is the nurse throwing up an appointment that she has already accepted.—ED. *T.H.*]

### UNSEEMLY AMUSEMENTS.

"AN INVALID" writes: Many will truly thank your correspondent (a "Lady Superintendent") for her most admirable and much-needed letter, where she draws attention to the additional suffering experienced by the inmates of our hospitals owing to the indiscreet and flighty behaviour of many of those who are appointed to nurse them in serious illness. The noisy amusements, so faithfully described, are, alas, not confined to one, but are spreading to many hospitals and asylums, changing the whole tone and demeanour which should surround and comfort the sick, forgetting that hospitals are houses of mercy, of quiet and comfort, built and maintained for the amelioration of suffering and the study of disease. For this purpose nurses are supposed to be there trained. Their life is not one of dull routine. They can go to theatres and other places of amusement, they have their clubs, their hours "off duty," generally a whole day free once a month, besides their annual holiday; therefore they are better off than many. Let all this be outside the walls of the hospital. To be allowed inside is an unfitness of things which should at once be stopped.

### GREEK LADIES AS RED CROSS NURSES.

MADAME F. L. PALLI (President of the Ambulance Department of the Greek Women's Union) writes from Athens: I have just read your article headed "Greek Ladies as Red Cross Nurses" in THE HOSPITAL "Nursing Mirror" of October 16th, and wish to reiterate my assurance to you that I made you in a letter some time ago now in reference to "An English Nurse's" letter of July 10th, that it contained nothing but what was absolutely true, and was neither gossiping, fault-finding, nor ignorant, but was a perfectly straightforward and impartial letter, and written without a malicious thought. Should your correspondent signing himself a "Knight of Justice of the Order of St. John of Jerusalem in England" carry out his threat, and attempt to injure the career of this nurse, I trust you will let me know at once, that I, with her numerous friends in Greece, Smyrna, and Alexandria (in all of which places she is well known and most highly appreciated) may come forward to support her. An English lady now staying with me, and who worked with the volunteer nurses and the *Daily Chronicle* nurses both at Volo and Lamien is leaving soon for England, and will be happy to give you any information you may care to have. You are at liberty to make what use you like of this letter.

### THE ROYAL BRITISH NURSES' ASSOCIATION. AN IMPARTIAL MEMBER.

NURSE LOUISA EAST writes: It is with a feeling of indignation that I read in your valuable paper of the doings of the so-called Members' Rights Defence Committee of the R.B.N.A., and of the insults that are offered to men of such unquestionable integrity and unselfish devotion to the best interests of our Association as the five members of the Executive Committee Mrs. Bedford Fenwick has been pleased to accuse of "general mismanagement of the affairs of the Association." And it is now time we effectually put an end to all this, and proved to these few dissatisfied members that they have not the support of the members in these accusations, and that they do not represent the feelings of the majority of the members. We have allowed these insults to pass unnoticed too long. But it is certainly not that we concurred with what was said, and the most effectual way we members who disapprove of the action of the so-called "Members' Defence Association," and of the unwarrantable insinuations and insults we have allowed our executive to bear unchallenged by us collectively, is to at once to sign a declaration stating that "we are perfectly satisfied that actions of the Executive Committee have been for the well-being of the Association, and we take this opportunity to assure them of our full confidence in them, and to thank them for all they have accomplished, and all they have borne so quietly for us." Again, I beg the members to consider well whom they wish to be their president—H.R.H. Princess Christian or Mrs. Bedford Fenwick—for it most certainly is the aim of the "Members' Rights Defence Association" to have in the end Mrs. Bedford Fenwick our president—if they can. We must prove our loyalty to our Royal President now by giving our unanimous "vote of confidence" in the Executive Committee.

### Where to Go.

ROYAL BRITISH NURSES' ASSOCIATION.—We are requested to state that another course of demonstrations in invalid cookery will be given during the winter months at the offices of the Association, 17, Old Cavendish Street, W. Many nurses will be glad to learn that the Association has again secured the services of Miss Earle, from the National Training School of Cookery, whose demonstrations last year were so much appreciated. Although arranged specially for the benefit of nurses, these lectures will be open to the public, and will be found very interesting and helpful to all who are perplexed with the daily problem so familiar in many households of tempting an invalid's appetite. The first demonstration will be given on Tuesday next, November 9th, at half-past two p.m. The terms will be as follows: Full course of ten demonstrations, members, 7s. 6d.; non-members, 12s. 6d. The tickets for the full course will be transferable. Single demonstrations, members, 1s.; non-members, 1s. 6d. Syllabus and further details can be obtained from the offices of the Association, 17, Old Cavendish Street, W.

DOWDESWELL GALLERIES.—A most charming collection of pastel views of Italy, Switzerland, and Germany, by Mr. Francis Chardon, are now to be seen at the Dowdeswell Galleries, 160, Bond Street. The variety and excellent choice of subject shown in these pictures is quite remarkable, and it is a genuine treat to have the opportunity of inspecting delightful scenes so skilfully depicted by this talented young artist.

BIRMINGHAM.—On Wednesday, November 24th, at eight p.m., a grand concert and entertainment will be given in the Masonic Hall, New Street, Birmingham, in aid of the Birmingham and Midland Hospital for Women. The Right Hon. the Countess of Dudley is patroness. Tickets, 2s. 6d. and 1s., at the out-patient department, Upper Priory.

THE MANCHESTER MIDWIVES' SOCIETY.—The tenth ordinary meeting of this society took place at half-past two p.m. in the Young Men's Christian Institute, 56, Peter Street, Manchester, on Thursday, November 4th. Meetings will be held at the same place at three p.m. on the first Thursday of every month throughout the season.

EASTMAN KODAK-PICTURE EXHIBITION.—At the New Gallery, 121A, Regent Street, London. Open to November 16th daily, ten a.m. to seven p.m. Photographs of the Royal family and enlarged photographs by many competitors are now on view. Luncheon and teas may be had.

### Appointments.

### MATRONS.

ROYAL ALBERT EDWARD INFIRMARY, WIGAN.—The new Sister elected on October 15th is Miss M. F. Mulvaney, who was trained for three and a half years, at the Children's Infirmary, Liverpool, and at the Royal Infirmary, Liverpool. She also held responsible posts in both institutions.

BRECKNOCK COUNTY AND BOROUGH INFIRMARY.—Miss Emily A. Hibberd, of the Cottage Hospital, Bocking, Braintree, Essex, was on October 21st elected Lady Nurse-Matron of the above infirmary. She has been, at different times, sister at the Royal South Hants Infirmary, Southampton; Salop Infirmary, Shrewsbury; Monsal Fever Hospital, Manchester; and Kensington Infirmary.

ST. PANCRAS SCHOOLS INFIRMARY, LEAVESDEN, HERTS.—Miss Lucy Lawrence has been elected Nurse-Matron of the above infirmary. She was trained at University College Hospital, where she has since held various appointments on the nursing staff.

### Minor Appointments.

KIDDERMINSTER UNION WORKHOUSE.—Miss Alice Barnes Jones was elected Superintendent-Nurse on October 26th, 1897. She had previously held appointments as matron of Swanage Cottage Homes and nurse at Dorset County Hospital. She was trained at Brownlow Hill, Liverpool.

ROYAL ALBERT EDWARD INFIRMARY AND DISPENSARY, WIGAN.—Miss H. B. Makin has been appointed Sister of the children's wards. Miss Makin was trained at the General Infirmary, Leeds, and was for two years sister at the same hospital, with charge of the ophthalmic theatre, and for a time acted as matron of the convalescent home connected with the Leeds Infirmary.

BIRMINGHAM AND MIDLAND HOSPITAL FOR WOMEN.—Miss E. A. Brander is the new Staff Nurse. Trained at Royal South Hants Infirmary, she afterwards studied gynæcological nursing at the New Women's Hospital, Euston Road, subsequently returning as out-patient sister to her old infirmary.

CITY FEVER HOSPITAL, BIRMINGHAM.—On October 22nd Miss Foster was elected Ward Sister of the above. Having completed her training at Staffordshire General Infirmary, she became charge nurse at the Infirmary, Birmingham, and subsequently at Smallwood Hospital, Redditch.

### Novelties for Nurses.

### CASH'S RUFFLED FRILLING.

THERE are few novelties that are likely to attain a wider or more enduring popularity than this useful and attractive article. Nurses above all other members of the community must recognise how much they owe to the famous Coventry firm which for so long has furnished them with the frilling that adorns their caps, but then it had to be gauged and regulated, which required skill in manipulation. Now this difficulty has been overcome by the production of a frilling already gathered for use, and which is procurable in different widths. The cambric is of the finest, and endless trouble will be saved by its employment. For trimming underlinen Cash's ruffled frilling will be found invaluable; it is very durable, excellent in quality, and requires but little trouble in application. It possesses the further advantage of washing well, and gophers into just the right folds. Our readers will be well advised to send for samples, when they will quickly recognise its beauty and utility.

### Notes and Queries.

**The contents of the Editor's Letter-box have now reached such unwieldy proportions that it has become necessary to establish a hard and fast rule regarding Answers to Correspondents. In future, all questions requiring replies will continue to be answered in this column without any fee. If an answer is required by letter, a fee of half-a-crown must be enclosed with the note containing the enquiry. We are always pleased to help our numerous correspondents to the fullest extent, and we can trust them to sympathise in the overwhelming amount of writing which makes the new rules a necessity. Every communication must be accompanied by the writer's name and address, otherwise it will receive no attention.**

*Probationers.*

(47) 1. Will you kindly tell me if I could get in any large general hospital in London any time I want to as a probationer free, (2) or as a paying one, and if the fee is very much; (3) and what examination do I have to pass? (4) Is 25 years the age to enter as a probationer at any general hospital?—*Annie Evans.*

(1) Probationers are appointed as vacancies occur. Apply to the matron of the hospital you wish to enter, and she will give you all information. (2) The fee for paying probationers varies. (3) A simple examination in reading, writing, and arithmetic, &c. (4) A very suitable age. You would find Honnor Morten's book, "How to Become a Nurse" (The Scientific Press, 28 & 29, Southampton Street, Strand; price 2s. 6d., post free), very useful.

*Prayers for Ward Use.*

(48) Could you tell me where I could get a book of the prayers for ward use? If you will kindly put the answer in "Notes and Queries" I should be grateful.—*Nurse T.*

Nurse T. has omitted to send name and address. Consequently, we cannot answer her query.

*A Cheap Suburb.*

(49) Can you tell me in which London suburb I could rent the cheapest house in a good locality? I would require one with five bed and three reception rooms, with small garden. Please state rent and taxes.—*Sister Flora.*

The query does not come within our province, but we are pleased to tell Sister Flora the best way to set about her quest. From a directory pick out the names of two or more respectable house agents in each suburb that might possibly suit, and write to them explaining requirements. Then visit such houses as they recommend, until the right one is found. This is troublesome, but it is worth while, as the suburbs differ so much in climate; for instance, North London is bracing, South relaxing. From our own experience, we think she will probably find a house at about £45 rent, and about one-third more must be added for taxes.

*Lady Doctor.*

(50) Will you kindly tell me where I must apply for full particulars of becoming a "lady doctor," and what the fees are and which training school is the cheapest?

The London (Royal Free) School of Medicine for Women, 30, Handel Street, Brunswick Square, W.C.; fee, in one payment, £125. The Edinburgh School of Medicine for Women, Surgeon Square, Edinburgh, N.B.; inclusive fee £95. The Medical College for Women, 30, Chamber's Street, Edinburgh, N.B.; similar fees to foregoing. The Glasgow School of Medicine for Women, Queen Margaret's College, Glasgow; fee about £100. Dublin: Full particulars from the Registrar, Royal College of Surgeons in Ireland. Schools of Medicine at Newcastle-on Tyne and Queen's College, Cork, open to women on the same terms as men.

*Examinations in Mental Nursing.*

(51) As I am in a position in an asylum where I have a good deal of spare time, I am anxious to continue the study of mental work. I should be greatly obliged if you could tell me if there is any other examination, for which I might prepare than that of the Medico-Psychological Association, as I already hold their certificate, and, if so, what books would be best to study? I have eighteen months' experience of hospital nursing, which I had to give up owing to ill health. I suppose there is no way of obtaining a nursing certificate other than by finishing my training in a hospital?—*Louie.*

There is no other examination. The course we should recommend is to finish up general nursing and obtain a certificate in that branch, and then revert to asylum work.

*Incurable.*

(52) I shall be obliged if you can give me any help or information as to the case herewith. I have a brother, age 30, who is of feeble mind, caused by a shock to the nervous system (by a convulsion) when he was an infant, from which he has never recovered, and we want to find either a private home or institution to place him in where he would be comfortable and well looked after. He would require attention in dressing and undressing, and cannot bath himself, otherwise he is not much different to any ordinary person, and is to some extent capable, though not able to earn his own living. If you could oblige me with any information to deal with such a case I shall be obliged.—*A. H. Daggett.*

Consult list of homes for incurables in "Burdett's Hospitals and Charities." If you have any difficulty in obtaining it, send to the Scientific Press, 28 & 29, Southampton Street, Strand. The price is 5s. post free.

*Anatomy and Physiology.*

*F. G. Rose* has not sent her address. We shall be pleased to answer her query when she does so.

*Justic.*—Please send names for private information only. This is our rule.

*Ayah's Home.*

Two correspondents have kindly replied to "E. Jackson's" query, and have sent the addresses of two homes—the Original Ayah's Home, 6, Jewry Street, Aldgate, London, E.C., and the Ayah's Home, 73, Bedford Road, Clapham, S.W.

## Figures and Tables

**Fig. 3. Fig. 4. f1:**